# Denouements of machine learning and multimodal diagnostic classification of Alzheimer’s disease

**DOI:** 10.1186/s42492-020-00062-w

**Published:** 2020-11-05

**Authors:** Binny Naik, Ashir Mehta, Manan Shah

**Affiliations:** 1 Department of Computer Engineering, Indus University, Ahmedabad, Gujarat 382115 India; 2 Department of Chemical Engineering, School of Technology, Pandit Deendayal Petroleum University, Gandhinagar, Gujarat 382007 India

**Keywords:** Machine learning, Support vector machine, Alzheimer

## Abstract

Alzheimer’s disease (AD) is the most common type of dementia. The exact cause and treatment of the disease are still unknown. Different neuroimaging modalities, such as magnetic resonance imaging (MRI), positron emission tomography, and single-photon emission computed tomography, have played a significant role in the study of AD. However, the effective diagnosis of AD, as well as mild cognitive impairment (MCI), has recently drawn large attention. Various technological advancements, such as robots, global positioning system technology, sensors, and machine learning (ML) algorithms, have helped improve the diagnostic process of AD. This study aimed to determine the influence of implementing different ML classifiers in MRI and analyze the use of support vector machines with various multimodal scans for classifying patients with AD/MCI and healthy controls. Conclusions have been drawn in terms of employing different classifier techniques and presenting the optimal multimodal paradigm for the classification of AD.

## Introduction

Modern technology has modified civilization in many alternative ways. Humans have nearly always been on a path of progression; however, due to technology, the twentieth and twenty-first centuries have seen many advancements that revolutionized individual^’^s work and living areas [1–3]. One of the spheres where technology has shown great development is in the field of machine learning (ML) [4–7]. ML is not just one technique or technology; however, it is a field of procedure science that includes various technologies to create systems that may learn from the information in their setting and then make predictions and perform actions once confronted with a replacement scenario [8–10]. ML is powerfully grounded in trendy arithmetic, drawing on experience in performing analysis, applied mathematics, set theory, chaos and dynamic systems, and calculus of variations among different areas [8, 11, 12].

One of the areas where ML is most important is healthcare. Healthcare is a part of life that we all consider to be something we are entitled to [13]. Healthcare is the diagnosis, treatment, prevention, and management of disease, illness, and injury and, therefore, the preservation of physical and mental well-being in humans. Mental health is a satisfactory functioning level of emotional and behavioral adjustments or absence of mental illness. Mental illness is a mental pattern that causes vital distress or impairment of personal functioning. Such features may be persistent, reversible, and remitting or occur as a single episode. Many disorders present signs and symptoms that vary widely between specific disorders. There is a large type of disturbance, and one of them is a chronic neurodegenerative disorder that typically starts slowly and worsens gradually, which is known as Alzheimer’s disease (AD).

AD is a general type of neurological disorder identified by continuous memory and cognitive decline. The disease is related to amyloid depositions and hyperphosphorylation of structural proteins that destroy the metabolic activity and lead to structural alterations in the brain [14]. Approximately 5.7 million American individuals have AD, and in 2015, AD was ranked as the sixth leading cause of death in the United States. In 2017, more than 16 million family members, as well as other unpaid caregivers, provided approximately 18.4 billion hours of care to patients with AD or other dementias. This care is valued at more than $232 billion and contributes as a factor of extended risk of emotional anxiety and negative mental and physical health issues of caregivers. However, a mathematical model estimates that early and accurate diagnosis of AD could help save up to $7.9 trillion in medical and care expenses [15]. Although, the indispensable treatment for AD has not been established yet, some medical and non-medical approaches can be used to reduce the developing symptoms based on the initial diagnosis of AD [16, 17]. The detection stage includes structural and functional neuroimaging techniques that allow interpretation of brain pathologies. One such technique is magnetic resonance imaging (MRI), which includes the utilization of strong magnetic fields and radio waves. Structural MRI (sMRI) is used to examine the anatomical irregularities of the brain that are induced by a traumatic event. In contrast, functional MRI (fMRI) is used to obtain a functional image of the brain based on blood flow and oxygen level. Its fundamental use is to collect pertinent data on the utilization of oxygen by the tissues. fMRI can trace the image of the brain’s functioning area by selecting the inordinate blood referred to as blood oxygen level dependence. Briefly, MRI provides anatomical structure of the brain, while fMRI provides metabolic performance of the brain. Single-photon emission computed tomography (SPECT) is one of the functional brain imaging techniques that provide knowledge on the regional cerebral blood flow (rCBF) and recognizes pathologic deviations in internal tissues and organs before the development of observable anatomical and structural changes [16, 18]. Positron emission tomography (PET) provides the rate of glucose metabolism using the tracer 18F fluorodeoxyglucose. The bilateral regions in the temporal and parietal lobes of the brain, posterior cingulate gyri, and precunei along with the frontal cortex and whole brain show a decrement in the rate of glucose metabolism in patients critically affected by AD [16, 19]. ML techniques are used in the identification of complex neuroimaging data as either the presence or absence of the disease in various neuropsychiatric disorders [20, 21]. Neural network, support vector machine (SVM), k-nearest neighbor (KNN) algorithm, ensemble, and regression models are the various ML techniques that are used to distinguish patients with AD and mild cognitive impairment (MCI) and healthy controls (HCs) [22–24]. Previously, there have been numerous efforts to evaluate the result using a monoclassifier, but due to some factors, satisfactory accuracy was not obtained, so various experiments were performed to evaluate the accuracy using a multiclassifier or ensemble method of the classifier [22, 25].

SVM is a multivariate, supervised data classification approach. Its main purpose is to provide the optimal hyperplane that divides data points of one class from another class [20, 26, 27].

The utilization of SVM techniques in neuroimaging scans has depicted the potential to predict future cognitive deterioration and transformation from MCI to AD [20, 28]. It can conceivably be employed in multimodal neuroimaging scans to improve the accuracy of AD diagnosis [20, 29, 30].

KNN is a nonparametric, lazy learning and feature similarity-based algorithm. It is an efficient algorithm for pattern recognition. It is a simple classifier, where data points are categorized based on the class of their nearest neighbor. KNN could probably be a good choice for a classification study where databases of high volume are used. Generally, medical databases naturally have a high volume; thus, KNN can effectively help predict the class of a new sample point. Studies have shown that the novel dimensionality reduction-based KNN classification algorithm outperforms the existing probabilistic neural network scheme in terms of the high average accuracy, sensitivity, specificity, precision, recall, and Jaccard and Dice coefficients and reduced data dimensionality and computational complexity [31].

The state-of-the-art ML approach of deep learning has exhibited notable execution over traditional ML approaches in classifying complex structures in complicated high-dimensional data. It is a promising diagnostic classification method for AD using multimodal neuroimaging data. Moreover, when neuroimaging data are limited, hybrid methods using deep learning approaches for feature extraction can yield better AD classification performance [32].

This study aimed to present the effects of using various technological advancements on AD diagnosis and assistance to patients with AD. The main focus of this study was to compare the accuracies of different ML classifier algorithms when applied to MRI scans for AD classification and further examine the diagnostic efficiency of SVM in distinguishing patients with AD from HCs based on different multimodal imaging scans, thus identifying the optimal combination of imaging modalities. This study also reviews two distinct case studies on AD diagnosis using ML techniques. The first case study depicts the significance of KNN and SVM classifiers with MRI scans, whereas the second case study shows the utilization of SVM classifiers in MRI and FDG-PET/rCBF-SPECT multimodal paradigm. Furthermore, the challenges and future scope of using such techniques have been discussed. Relevant conclusions have been made regarding applying different ML classifier techniques and presenting the optimal multimodal paradigm for the classification of AD.

## Impacts of technology in assisting patients with AD and AD diagnosis

Technology has various potential applications in the treatment and assistance of patients with AD. These include the socially assistive robots (SARs), global positioning system (GPS)-based tracking of patients, use of sensors and wearables, ML algorithms in the detection and diagnosis of AD [33, 34].

Traditionally, in the absence of technological methods, individuals with AD were cared for by their family members. Reviews show that 68% of caregivers of patients with dementia have burden and stress in the procedure of caregiving and 65% develop symptoms of depression. Alternatives to augment care with the use of robots can help reduce this stress. There are SARs: Robots that are trained to assist patients with AD by the means of social interactions are used [35, 36], and the rehabilitation process that they follow involves the crucial factor of assessing the patient’s movement rate and intensity, associated with their involvement with the robots [35, 37–39]. Besides SARs, there are some humanoid robots that have activity monitoring functions, which learn by matching algorithmic parameters with a gesture viewed through its camera and repeatedly executed by different persons to permit variations. Aberrations from the set parameters can hence be identified as abnormal, and signals are issued. The robots are trained to follow a technique in which they detect whether a person forgets to take their medication at the prescribed time and thus perform necessary actions, such as investigating their state of current mental state, generating necessary alerts and reminders to the patient or even notifying relatives about the patient’s condition in case of danger. Despite the potential advantages that the robots can serve to assist and help alleviate the problems related to patients with AD, it has several implementations and ethical limitations in its use. The major barriers pertaining to the adoption of SARs can be an inconsistency between the requirements and solutions provided by the robots, some factors related to service and usability, and lack of familiarity with technology [40]. The reason behind the reluctance toward the use of humanoid robots in the care of elderly patients is the robot appearance [41–43]. Other common factors that hinder the implementation of robots is the area that it occupies due to the size of its system [44, 45], robot bearing an adverse portrayal of proposed users because of a stigmatizing aesthetic [46, 47], and some safety issues, such as lack of faith in the robot [44]. Six chief ethical issues related to the use of robots in assisting elderly individuals include (1) deception and infantilization; (2) loss of individual freedom; (3) situations in which elderly individuals should be permitted to manage robots; (4) likely decrease in the duration of human associations; (5) increase in the feelings of objectification and lack of control; and (6) loss of privacy [48].

Disorientation and wandering are the most common traits shown by an individual with memory and cognitive impairment. A patient with AD may not recognize their location and identity or the time and date. Presently, a technology known as GPS is used to accumulate data on the spatial motion of individuals. The primary function of the GPS receiver is to receive the signal that is transmitted by the GPS, which is used to determine the exact position by the amalgamation of four different signals from the satellite [49]. It tracks the patient’s regular routes, characterized by frequent destinations, in a graph form. Then, a developed algorithm studies the deviations from usual paths and sends out alerts to help the user get back on track. The major factor that limits the use of GPS technology in assisting patients with AD is the accuracy that it provides. The GPS signals weaken and are distorted when some obstacles such as walls or buildings come in its path. This even makes the GPS incapable of indoor positioning [50]. Additionally, patients with advanced dementia will be incapable of remembering to carry the monitoring device or handle it in case it is misplaced [51].

Apart from these, a technology powerful in communication and computational assistance is used to detect psychological alterations and psychomotor agitation using portable devices, such as wearable individual monitoring systems. A notable evolution has been found in the field of wearable sensors, which works on the mechanisms that integrate the sensors with wireless communication, thereby integrating the sensors in tissues [52]. Before these acquired signals can be employed in the detection of psychomotor agitation, they need to be preprocessed to remove unnecessary disturbances [52]. This primarily necessitates the use of ML. Feature selection is performed on the acquired signals instead of using the raw signals by utilizing classifiers, such as Naive Bayes, decision trees, discriminant analysis, SVMs, and KNN. To detect or record abnormal activities of patients with AD, an integrated technique of wearable systems, sensors, and ML that reduce the number of features that are extracted from the acquired signals can be employed. However, wearable technology alone shows barriers in application with product and design-related issues. Capturing early-stage activities becomes essential for developers to address these issues. Other factors that limit the use of wearables include the need to clean and maintain the devices regularly and replacement of the batteries at appropriate periods [51].

However, the barriers shown by various technologies can be overcome using ML techniques. AD demands an early and efficient diagnostic process. This can easily be facilitated using ML classifiers. These classifiers follow effective and powerful algorithms that work on the principle of learning. Contrary to other technologies, ML provides assistance and treatment of AD with ease-of-use and ensures good accuracy results. These results are reliable and secure. The chief barrier regarding the security and privacy of the patients that existed in the use of other technologies is removed by the introduction of ML techniques in AD diagnosis and treatment.

ML classifiers, such as SVM, KNN, and Naive Bayes classifiers, can also be employed to provide optimal AD diagnosis results by combining neurofunctional and neurostructural scans such as MRI, PET, SPECT, and cerebrospinal fluid (CSF) analysis in different combinations.

## Comparing the use of various ML classifiers in the diagnosis using MRI

Zhou et al. [53] recommended the combination of MRI and neuropsychological tests and the mini-mental state examination (MMSE) in the classification of AD using an SVM. The study consisted of 59 patients with AD, 127 cognitively normal (CN) individuals, 67 patients with amnestic MCI, and 56 patients with nonamnestic MCI. MMSE scores play a vital role in discriminating patients with AD from normal individuals. Image analysis is conducted using FreeSurfer to produce volumetric variables. The study was twofold; that is, the data were divided into two parts: one part for training and another part for testing. To limit the error percentage in detecting the accuracy of the proposed method, it was implemented 50 times, and the overall average accuracy was calculated for the accurate result. An SVM classifier was used in the study along with kernel function to build a maximum margin classifier. The combination of the MMSE score and volumetric MR images shows an improvement of 10% in the accuracy rate, and the analysis displayed an accuracy of 92.4%.

Zhou et al. [54] suggested a method for classification of AD from the HCs based on the MR images using the Naive Bayes classifier by selecting wavelet entropy as the selected feature. Wavelet transform is a function that aimed to represent the image into multiple scales, such as one and two dimensions by filtering the original MR images and representing the image along the x and y orientation. The wavelet transform is used in each dimension to obtain the detailed segments of the MR images. A total of 64 subjects (18 HCs, 46 patients with AD) underwent T2 MRI for the classification methodology. Thus, the detection rates in the classification of patients with AD and HCs was 92.60%. Although the flaw of the proposed model is that the interpretation of the wavelet entropy is difficult, multidisease classification could be one of the areas for advancement.

Belmokhtar and Benamrane [55] intended to create a method for discriminating between AD, MCI, and CS by combining multiple binary SVM classifiers based on whole-brain voxel-based morphometry analysis applied to MR images in the OASIS database. The selection of features is performed using the VBM technique [56] along with the MMSE and Clinical Dementia Rating (CDR) tests for improving the AD detection rates. Moreover, the Java Agent Development Framework is used to decrease the classification time. To estimate the performance of each binary SVM model, fivefold validation was implemented; that is, test data were obtained from five subjects, while training data were obtained from the remaining 25 subjects, and finally, the average of all SVM models was calculated as a result of 100% accuracy for classification of patients with AD.

Ali et al. [57] suggested a novel classification technique TANNN in MRI based on the module of preprocessing using filtration and segmentation using content-based image retrieval. Feature extraction is conducted by analyzing the threshold and disease classification to identify the AD shape in MRI and classification time and accuracy of the different classifiers. The OASIS dataset was obtained in the study, which consisted of 416 images from individuals aged ≤ 18 years, for estimation and comparison. The result of the study was that the decision tree had the best accuracy of 96.19%, but the KNN proved to be more appropriate in terms of detection rate and classification time. From this study, TANNN appears to be important for real-time classification because of its high accuracy rate.

Rueda et al. [58] introduced an image analysis that is based on selecting the salient brain patterns for classification. This classification is not about the specific salient points but selecting the whole region as a salient region. This image analysis is capable of mapping any brain region that has been already associated with brain conditions, which is validated using two datasets, that is, MIRIAD and OASIS in four groups of patients with AD. The good deed of the model is that the model can be interpreted as patterns are mapped into the brain images and used to determine the significance of the selected region for the classification of patients with AD and HCs. Salient brain patterns in the classification technique displayed better performance than the traditional feature-based morphometry, and such a technique has not been studied in characterizing and classifying patients with AD based on MR images. The accuracies of the four groups were 86.05%, 80.16%, 76.47%, and 70.2% for G1, G2, G3, and G4, respectively.

Aruchamy et al. [59] proposed a two-stage method for detection in brain MRI: the first stage involves preprocessing, segmentation, and skull stripping of MR images accompanied by the application of various fractal analysis techniques, fractal Brownian motion, box-counting method, and differential box-counting method, and then one fractal analysis technique was selected based on the results. Image preprocessing utilizes the technique contrast limited adaptive histogram, which intensifies the MR images by increasing the contrast [60], which is performed by splitting the MR image into small equal size boxes and then performing the histogram equalization technique in each box. The fractal analysis technique is used to differentiate between normal and abnormal conditions. In the box-counting analysis, the MR image is overlaid with boxes, whereas, in differential analysis, the image is split into a smaller size grid. The accuracy of box-counting analysis was 85.287%, which is better than the differential box-counting method accuracy of 76.725%. The fractal Brownian motion accuracy was 88.774%, which is more reliable than those in both analyses.

Zhang et al. [61] developed a new ML classification system based on eigenbrain for establishing a computer-aided diagnosis (CAD) technique for the detection of AD. First, preprocessing was performed on the volumetric data, all data were motion corrected, and thereafter, a 3D MR image which was then normalized to the Talairach coordinate space. CDR score was obtained as a label for quantifying the intensity of the symptoms of AD [62]. In the assessment of the patient, six different areas were selected, including personal care, memory, home and hobbies, orientation, community affair, judgment, and problem-solving. In the study, the subjects with a CDR score of 0 were recognized as normal controls (NCs), whereas the subjects with a CDR score > 0 were identified as patients with AD [63]. Visual representation of eigenbrain was utilized to differentiate between patients with AD and NC subjects. The SVM classifier was employed in the study, which was trained because of its simplicity and fast speed with the help of Sequential Minimal Optimization [64]. Kernel function was applied using the kernel SVMs (KSVMs), which expand the linear SVM to a nonlinear SVM classifier to substitute the dot product form in the SVM classifier [65]. The results showed an accuracy of 92.36%.

Beheshti et al. [66] introduced an automatic CAD system utilizing the sMRI data based on the feature ranking mechanisms for the identification of AD. The selected system consists of four stages, and the purpose is to recognize the variations in the gray matter as the volume of interest, and so the variations in the gray matter of AD were compared to those of HCs with the help of a voxel-based technique. In the second step, the voxel intensity values of the volume of interest are drawn as features. Further, in the third step, these selected features are then ranked based on seven feature ranking methods, namely, Gini index, mutual information, Pearson’s correlation coefficient, Fisher’s criterion, information gain, statistical dependency, and t-test score. The classification is carried out using the SVM classifier in the last stage. For the investigation, a total of 260 Alzheimer’s disease neuroimaging initiative (ADNI) datasets were examined with tenfold cross-validation. The outcome of the study was 92.48% for the classification of AD. The result of the study shows that the execution of the utilized system is analogous to the state of classification methods.

Salvatore et al. [67] proposed an ML technique that extracts the multivariate biomarker from the sMRI for the classification of AD. In the study on 162 NC patients, 76 MCI patients who convert to AD within 18 months, i.e., MCI convert (MCIc), 134 MCI patients who did not convert to AD within 18 months, i.e., MCI non convert (MCInc), and 137 patients with AD were taken into consideration. T1-weighted sMR images, which underwent geometry correction and intensity correction, were obtained. As a part of preprocessing by coregistration, spatial normalization was conducted in all MR images, which included various steps, such as image re-orientation, cropping, skull stripping, and normalization. Each image was divided into the gray matter and white matter, which were then smoothed with the help of Gaussian kernel. The classification steps include two phases: feature extraction from the MR images with the help of principal component Analysis amalgamated with Fisher’s discriminant ratio was executed in the first phase, and in the second phase, each subject was classified based on the predictive model, which is utilized in classifying the subjects. The implemented classifier manifested an accuracy of 66% for MCIc vs MCInc, 76% for AD vs CN, and 72% for MCIc vs CN.

Westman et al. [68] exhibited a multivariate tool orthogonal partial least square for determining the type of normalization, which is more suitable for different parts of MRI, to determine the best combination that yields the highest accuracy for classifying patients with AD. This multivariate tool was implemented on 699 subjects (AD, 187; MCI, 287; and CTL, 225), which were obtained from the ADNI database. Preprocessing of the MR images was conducted using mean centering and unit variance scaling, so the data are more understandable, and images were repositioned around the origin as the data were three-dimensional. This multivariate tool was able to provide 91.50% accuracy in the classification of patients with AD and HC but 75.90% accuracy in the classification of patients with MCI and AD. This study also suggested that the combination of raw cortical thickness and subcortical volumes yielded the best detection accuracy rates for distinguishing patients with AD from HCs.

Islam and Zhang [69] presented a convolutional neural network (CNN) model and framework that classifies three stages of AD by analyzing the MR images based on various hyperparameters. The OASIS data of 416 subjects aged between 18 and 96 years were collected to implement the CNN. The model is based on the Inception-v4 network, which includes several convolution layers, and the pre-processed MR images are obtained as an input and passed through the stem layer from which the data are passed to the convolution layers in the flow as Inception-A, Reduction-A, Inception-B, Reduction-B, Inception-C, and Reduction-C. The data of all these layers are collected and combined and then put into the softmax layer, which classifies the data into non-dementia and very mild, mild, and moderate AD. The current accuracy of the method is 73.75%, which displays better accuracy than those of all traditional methods previously used. The proposed CNN demonstrated faster performance to classify the brain MRI for AD.

Gulhare et al. [70] proposed a deep neural network (DNN) classification for studying the MR scans of AD, MCI, and HC. MR images are preprocessed and segmented to exclude the information that is not suitable for better understanding and select different features from the segmented images. Several attributes are extricated from the image, and then DNN classification was applied to the MR images for efficient and accurate results. The ventricle region was selected as an attribute using the watershed method. The DNN having several hidden layers is introduced to the extracted images for classification. The study outcomes showed that DNN displayed the highest accuracy of 96.6% for specific pairs of selected attributes compared to 90.3% when all the attributes were selected. The study proved the utility of the DNN classifier, which is better than the SVM classifier [71].

### Comparative study

Zhou et al. [53] used SVM for the classification combined with variables affecting classification along with the MMSE score that was selected rankwise. However, all possible combinations of variables were not selected, and thus a combination might be ignored, which could have provided better results. Zhou et al. [54] used a single wavelet entropy transform along with Naive Bayes classifier. This proved to be slightly more accurate and less complex than the study of Zhou et al. [53]. However, it did not prove to be powerful for multidisease classification because the interpretation of the wavelet transform was difficult and different brain pathologies were considered as single abnormal.

Belmokhtar and Benamrane [55] used a binary SVM with voxel-based morphometry and Java Agent Development Framework to analyze the classification process and minimize the computational time, respectively. As a result, the accuracy obtained was 100%, but it varied according to the change in the number of MRI datasets. In addition to such techniques, Ali et al. [57] proposed the use of TANNN for the microlevel classification pattern finding. This proved to be more suitable because of its faster execution rate, but it could be more robust if it had the capability of mining the different components of the image like shape and texture. A similar pattern-finding method was also proposed by Rueda et al. [58], in which the bottom-up and top-down approaches were used to extract information. However, this approach was limited to a localized difference and failed to discover a complex interrelation among these local differences. In contrast, fractal analysis proposed by Aruchamy et al. [59], not only could find changes in the internal structure but was also precise and time efficient. Fractal Brownian motion was more accurate than the other two fractal analysis methods as it was measured by calculating the difference in pixel intensity of row and column pixel pairs.

Zhou et al. [54] proposed CAD in which the eigenbrain for the selected slice of 3D data was classified using KSVM and particle swarm optimization for better results. However, Beheshti et al. [66] proposed an automatic CAD using the statistical feature ranking selection that proved to minimize the error. All of such brain pattern-finding approaches failed to detect the spatially distributed pattern of brain anatomy. Thus, the need for the multivariate tool for finding the pattern was considered.

Salvatore et al. [67] and Westman et al. [68], both proposed the multivariate tool. Salvatore et al. [67] extracted the spatially distributed biomarkers from the MRI, whereas Westman et al. [68] included 259 variables for the orthogonal partial least squares to latent structures (OPLS) analysis. The combination of regional cortical thickness and cortical and subcortical volumes proved to be more accurate.

In the last 15 years, the amount of data generated is increasing exponentially, which is also known as big data. The rise in data leads to replacement of traditional ML algorithms with deep learning. Deep learning has taken over the traditional ML methods because of its capability to learn from its hidden architecture of any complexity.

Islam and Zhang [69] implemented the deep CNN model for the classification of AD. This work could be more enhanced by transfer learning and exploring more hidden convolution layers, whereas the DNN proposed by Gulhare et al. [70] using the Niblack thresholding algorithm proved to be more accurate, having an accuracy rate of 96.6%.

Table 1 represents a brief review of similar studies conducted using various ML classifiers for AD diagnosis based on MRI scans. However, using only one imaging modality cannot serve the purpose of classification as every modality has its own strengths and shortcomings, and so for optimizing the models and making them more robust, the need for multiple modalities increases.

**Table 1:** Use of different ML classifiers for AD diagnosis using MRI scans

Technique	Classifier used	Modality	Data	Accuracy	References
Region growing	Artificial neural network	MRI	KHMC	(100%)	[72]
3D inception	CNN	MRI	ADNI	AD/NC (93.30%) AD/MCI (86.70%) MCI/NC (73.30%)	[73]
Fractal analysis	KNN, SVM (linear), SVM (RBF), HLP (polynomial)	MRI	OASIS	SET-1: KNN (61.76%), SVM (linear) (59.41%), SVM (RBF) (64.71%), HLP (polynomial) (65.29%) SET-2: KNN (72.31%), SVM (linear) (75.38%), SVM (RBF) (76.15%), HLP (polynomial) (86.15%) SET-3: KNN (59.00%), SVM (linear) (64.00%), SVM (RBF) (68.00%), HLP (polynomial) (67.50%)	[74]
Salient brain patterns	SVM, NN	MRI	OASIS	SVM (84.21%) NN (65.78%)	[75]
K-OPLS, OPLS	Multivariate data analysis	MRI	ADNI	K-OPLS (88.70%) OPLS (88.40%)	[76]
Hippocampal shape feature	SVM	MRI	ADNI	CASE 1 (90.40%) CASE 2 (89.40%) CASE 3 (90.40%) CASE 4 (93.60%)	[77]
ROI	Naïve Baye, SVM, KNN	MRI	OASIS	Naive Baye (90.00%) SVM (95.00%) KNN (95.00%)	[78]
Hippocampus volume, tensor-based morphometry, cortical thickness	LDA	MRI	ADNI	HC vs AD (89.00%) HC vs P-MCI (84.00%) S-MCI vs P-MCI (68.00%)	[79]
Multivariate techniques	Logistic regression	MRI	Self	AD vs HC (83.00%)	[80]
Gray-level co-occurrence matrix	Adaboost, KNN	MRI	OASIS	AD vs NC: Adaboost (100%), KNN (92.75%) AD vs MCI: Adaboost (100%), KNN (92.31%) MCI vs NC: Adaboost (90.28%), KNN (83.33%)	[81]

Authors assume that SVM could be better than the other classifiers as SVM can easily provide more reliable search accuracy for image classification. It uses a mechanism known as kernels, which determine the distance between two objects almost accurately. SVM is also robust against overfitting. It proves to be efficient in situations where the number of dimensions is higher than the number of samples.

## Comparing diagnostic efficiency of various multimodal scans using SVM classifier

Dukart et al. [82] employed a classification algorithm utilizing FDG-PET and MRI data for two distinctive datasets. Two different datasets, such as Leipzig and ADNI datasets, were obtained and then compared for better accurate rates. Moreover, the number of subjects of ADNI was more than Leipzig, so the number of subjects was restrained to evade classification inclination toward ADNI. Preprocessing of the images was performed by incorporating PET and MRI images to a resolution of 1 mm^3^ × 1 mm^3^ × 1 mm^3^. Gaussian kernel was used to smooth the images as a part of preprocessing, followed by excluding the voxels in the images for obtaining the binary mask. Finally, all images were normalized, and SVM was applied to the modalities. Multiple modalities displayed better accuracy rates than the single modality for both the datasets, that is, 85.7% and 100% for ADNI and Leipzig, respectively. Moreover, the accuracy of the combined modality is 90% for the combined dataset of ADNI and Leipzig subjects.

Dinesh et al. [83] introduced a novel approach based on nonnegative matrix factorization (NMF) and SVM for the CAD in determining AD. The classification is performed with the database of fMRI, PET, and SPECT images. The images are labeled based on information stored in each voxel of the brain. The relevant information is labeled as AD, whereas the information that is not relevant is labeled as NOR. Furthermore, on the labeled voxels, Fisher’s discriminant ratio is used for the feature selection variables from the voxels, and then NMF is used for feature reduction for the fMRI images where all the selected variables contain only positive values, followed by the application of the SVM classifier for determining the detection rates for AD. The NMF-SVM approach generated an accuracy of 91% in classifying patients with AD for both datasets.

Dyrba et al. [84] examined the multimodal data received from the 53 subjects, from which there were 28 patients with AD and 25 HCs. The multimodalities included in the determination of the classification of AD were DTI, gray matter, and resting-state (RS) fMRI. SVM as a classifier was included in the process of determining and comparing the accuracy of the single modalities vs. multimodalities. The areas under the curve (AUCs) were 87% for the DTI, 86% for the gray matter, and 80% for the RS fMRI, while the combination of the modalities manifests an AUC of 82%, which did not display any major difference compared to the single modality. Further, the average ratios of SVM were approximately 52% for GM, 67% for RS-fMRI, and 99% for DTI.

Westman et al. [14] utilized a multivariate analysis OPLS for classification of the AD from the CTL and MCI by combining MRI and CSF on the dataset of 369 subjects classified as follows: 162, MCI; 96, AD; and 111, CTL. Preprocessing is performed using the FreeSurfer pipeline to generate the regional cortical thickness and subcortical volumetric measures, followed by the use of watershed to eliminate the non-brain tissue that is not relevant to the study [85]. The modalities MRI and CSF were combined for the classification of AD from CTL and MCI from CTL. The combined modalities manifested better accuracy compared to the performance of the single modality, and the accuracy of the combined modality was approximately 91.8% for the classification of AD vs CTL. Regarding MCI vs CTL, the accuracy decreased to 77.6% at baseline. Thus, the approach of using combined modalities in the multivariate model for detection of AD was beneficial compared to a single modal approach for distinguishing AD from CTL.

Liu et al. [86] suggested a multi-task training that works on the principle of selecting the features from MRI and PET images and thereby maintaining the intermodality link after the prediction of the selected vectors. The feature selection technique that includes the intermodality link is generated by operating the technique as a multi-task regression problem followed by the application of a multi-KSVM method to unite the features selected from the PET and MRI. Based on the selected features, the kernel is determined by the application of SVM, followed by combining all corresponding linear kernels to form a multi-KSVM for various modalities. The methodology defeated the compared method in distinguishing AD from NC and MCI from NC with accuracy rates of 94.37% for AD classification and 78.8% for MCI classification.

Young et al. [87] presented a Gaussian process to classify AD from MCI. This process works by selecting features in the single modality and thereby finding the corresponding kernel and combining the various kernels of MRI, PET, and APOE genotype, forming a mixed kernel, and lastly utilizing the GP classifier for classification accuracy. The outcome of the Gaussian process ranged from 0 to 1, which depicted the determined probability that a subject is classified to which group, followed by binarizing these outcomes with the help of threshold. The outcomes of the Gaussian process classifier are then compared with those of SVM. The Gaussian process classifier displayed 74.1% accuracy for the combined modalities (MRI + PET + APOE) but 67.8% accuracy for the SVM classifier, suggesting that the application of the Gaussian process is better than SVM for multimodal classification.

Davatzikos et al. [88] studied the patterns of MRI combined with the CSF for the classification of AD along with MCI. For the study, data of 239 patients were examined, and based on the CDR, the MCI dataset was split into two parts: converters (MCIc) and non-converters (MCInc). The MR images consisted of T1-weighted images, which were acquired using volumetric 3D MPRAGE, which then underwent calibration, correction, and skull stripping. Further CSF samples were included with the help of ADNI. The first step for image processing was to align the anterior commissure plane; then, cerebellum tissues and skull were removed. The images were then divided into ventricles, gray matter, sulcal CSF, and white matter. In the study, SPARE-AD was employed as a biomarker as it manifested better prediction for the conversion of MCI to AD [89]. The score of SPARE-AD for the classification of AD and CN was in the predicted range. Weka software was used with the CSF biomarkers and SPARE-AD as inputs for the evaluation. Subsequently, a linear SVM was employed in the framework. The results manifested that the biomarker showed a slightly less predictive result compared to SPARE-AD.

Vemuri et al. [90] presented a study that showed that the implementation of the SVM classifier on an individual sMRI compared to more scans can provide essential data for classification of AD. This study aimed to train a model for classification of AD and CN based on MRI scans of 280 subjects along with the genetic and demographic information. The MR images were executed on 12 different scanners, which underwent standard quality calibration of all axes. The model for classification was developed in a hierarchical manner. Model I includes the utilization of the SVM classifier for classifying 280 training datasets of sMRI as an output structural abnormality index (STAND) score was produced. Furthermore, in Model II, the STAND score was augmented by including demographic information and also the known genotype for increasing or decreasing the risk of generating AD. APOE is also included in Model II. Lastly, in Model III, the tissue density and demographic variables were decreased. As a result, the STAND scores of Model II and Model III were 88.5% and 89.3% respectively.

Ritter et al. [91] examined the foresight of MCI to AD based on multimodal data. For the study, all patients diagnosed with NL or MCI until the age of 3 years were included in the MCI stable group, whereas the patients who had progression to AD within 3 years were classified as the MCI converter group. A total of 237 patients were included: 151 in the MCI stable group and 86 in the MCI converter group. Furthermore, for the study, features were selected from 10 different modalities (total of 288 features). PET, CSF, and MRI were some of the modalities from which features were obtained. Then, the selected features were classified with the help of the SVM classifier, and the performance was compared with random forest and single classification tree. To compare the performance between all three classifiers, cross-validation was performed. First, the data were divided into three parts, and the other two parts were the validation parts. Based on the validation parts, a tenfold cross-validation was executed and repeated 30 times to obtain more stable results. Based on the modalities and selected features, SVM outperformed all classifiers in terms of accuracy.

Zhang et al. [92] implemented the multi-modal methodology for classification of AD, MCI, and HCs by combining MRI, PET, and CSF on the ADNI database (51, AD; 99, MCI; and 52, HC). Image preprocessing and feature extraction are conducted before the addition of the SVM. Preprocessing is a process of correction of images in the anterior commissure and posterior commissure, followed by skull stripping using the brain surface extractor [93]. After the pre-processing and skull stripping is performed, the images are segmented into gray matter, white matter, and CSF. A total of 93 regions of interest (ROIs) are selected as a feature of MRI and PET images, and features selected for the CSF are their original values. The outcome of the classification methodology was 93.2% when all three modalities were combined, while the single modality manifested the highest classification rate of 86.5% for AD. Likewise, for MCI, the classification accuracy rate was 76.4% for the combined modalities but only 72% for single modality.

Kavitha and Thyagharajan [94] focused on the application of combined images of MRI and PET or SPECT for classifying AD from the normal conditions using the binary SVM classifier based on feature selection and determining the accuracy of the combined images and comparing them to the single modality. In this study, a total of 120 images were used from which 86 were used for training purposes, while 34 were used for testing purposes. Preprocessing involves the process of removing the artifacts from the images. Images were combined with the help of the redundant discrete wavelet transform. The outcomes of the study manifested classification accuracy of 94.1% for the fused images, which was better than the classification rates of the single modalities.

### Comparative study

Ritter et al. [91] predicted the conversion of MCI to AD using multiple modalities involving MRI, PET, and CSF. The imputation was performed with the help of the EM algorithm. The features were selected by automatic and manual selections. SVM was used as a classifier to train and test the data. The replacement of missing data, manual selection of the features, and training and testing on the same data are considered limitations because the manually selected features vary according to the level of expertise. Compared to that in the study by Zhang et al. [92], extracted features from the MRI, PET, and CSF were individually fed into the kernel matrix. For the classification, all three individual kernel matrices were fused into one kernel combination. As the study of Zhang et al. [92] did not involve any missing value and manual feature selection, it proves to be more accurate and reliable than the study of Ritter et al. [91].

Among all discussed studies involving SVM based multimodalities, the study by Kavitha and Thyagharajan [94] can be considered more accurate and reliable because it has the minimum false-positive error rate compared to other multimodal techniques. The utilization of the redundant discrete wavelet transform technique to combine the different brain modalities into one proved to be beneficial as it manifested an accuracy rate of 94.1%.

Table 2 below shows a brief review of similar studies conducted using SVM classifier for AD diagnosis based on multi-modal imaging scans.

**Table 2:** Use of SVM classifier for AD diagnosis using different multimodal imaging scans

Technique	Classifier used	Modality	Accuracy	References
Pattern recognition	Convolutional neural network and sparse autoencoder	MRI and PET	AD vs NC: 3 × 3 × 3 (90.30%), 5 × 5 × 5 (91.10%), 7 × 7 × 7 (89.80%) MCI vs NC: 3 × 3 × 3 (87.90%), 5 × 5 × 5 (89.10%), 7 × 7 × 7 (89.20%)	[95]
Pattern recognition	Random forest algorithm	MRI, PET, CSF, and APOE	AD vs NC (91.80%) AD vs MCI vs NC (60.20%) MCI vs NC (79.50%)	[96]
Pattern recognition	Multi kernel learning	sMRI and DTI	AD vs NC (90.20%) MCI vs NC (79.42%) AD vs MCI (76.63%)	[97]
Whole-brain parcellation	SVM	MRI and DTI	Multimodal with 73 features (72.40%) Multimodal with 15 univariate features (72.11%) Multimodal with 15 multivariate features (99.60%)	[98]
Multi-task feature selection	Multi KSVM	MRI and PET	AD vs NC (94.37%) MCI vs NC (78.80%)	[99]

Authors interpret that multimodal imaging techniques are favorable than single modality techniques as they play a crucial part in the classification of ADs by empowering the clinician skill to provide better outcomes by performing screening, monitoring, prediction, outlining, treatment supervision, healing efficiency, and recurrence prediction. Moreover, accuracy of the multimodal imaging techniques are improving and becoming more reliable than that of single modality techniques when it comes to classifying patients with AD.

## Case studies

### Case study 1: classification of AD using KNN and SVM based on selected slices from 3D MRI

#### Dataset

Gad et al. [100] presented the classification accuracy of KNN and SVM modalities using selected slices from 3D MRI. The study used data of 120 subjects aged 57–91 years. These 120 subjects included 40 HCs, 40 patients with MCI, and 40 patients with AD. These data were procured from the National Alzheimer’s Coordinating Center.

#### Preprocessing

Next, preprocessing was performed to ensure high accuracy. Ten slices of the brain that consisted of the highest information were extracted from all subjects by 3D-T1 MRI and used. The selected slices were separated to remove noise; then, all datasets were normalized by applying the ROI to exclude the black space outside the brain.

#### Feature extraction

Feature extraction is a process of making data compact, redundant free, and significant. Following the feature extraction process, few features were selected from the dataset for finding the optimal subset of features that increases the efficiency by decreasing the measurement cost of the learning algorithm and used in the training method of the learning algorithm. Different features considered in the study were mean, entropy, energy, contrast, correlation, homogeneity, skewness, kurtosis, total brain area, total black area, gradient mean, and image symmetry.

#### Classification

Classification of the images provides the knowledge regarding the appearance of abnormality in the brain image that is to be trained and tested by distinguishing the various abnormal brain images based on an optimal feature set.

#### SVM classification

For nonlinearly separable patterns, SVM kernels are used. The SVM classifier works in two steps. The first step includes distinguishing HCs from abnormal controls, and the second step includes further classifying the abnormal controls into patients with either MCI or AD using the polynomial kernel function according to the following equation [100, 101]:

$$ \mathrm{k}\left(x,y\right)={\left({x}^ty+i\right)}^p $$

where *x* and *y* are two feature vectors and *i* is a free parameter trading off the influence of higher-order vs lower-order terms in the polynomial.

#### KNN classification

Unlike SVM, KNN uses only one-step classification procedure to distinguish all subjects as either HC or MCI or AD. The key strategy of this method includes classifying unlabeled testing data points based on the K value, i.e., the number of nearest neighbors to the testing example. The testing set examples can be classified by various distance measures, such as Euclidean and Riemannian. This review depicts the use of Euclidean distance for determining the nearest distance [100, 102].

The Euclidean distance formula is as follows:

$$ \mathrm{d}=\sqrt{\sum \limits_{i=1}^n{\left({x}_i-{y}_i\right)}^2} $$

where *x*_*i*_ and *y*_*i*_ are two feature vectors used in classification using a variable number of neighbors to classify all subjects with different values for K = 4, 5, 6, 7.

#### Results

Twelve features were extracted from 10 selected slices for each slice. Subsequently, permutations and combinations of these features were applied to both classifiers separately, to find the best accuracy for each class. The 120 subjects were bifurcated as 72 training subjects and 48 testing subjects.

The training subjects were further divided into 24 HCs, 24 patients with MCI, and 24 patients with AD. The testing subjects were further classified into 16 HCs, 16 patients with MCI, and 16 patients with AD. For the KNN classifier, the value of K varied, and data were tested for 212 permutations of all features, and then the best number of features was selected that have the highest accuracy for each class. For the SVM classifier, a total of 212 permutations of all features are examined for different values of SVM polynomial order, and ultimately the most suitable combination of features having the greatest accuracy has been selected for AD, HC, and MCI for the polynomial orders 3 and 4.

**Fig. 1:**
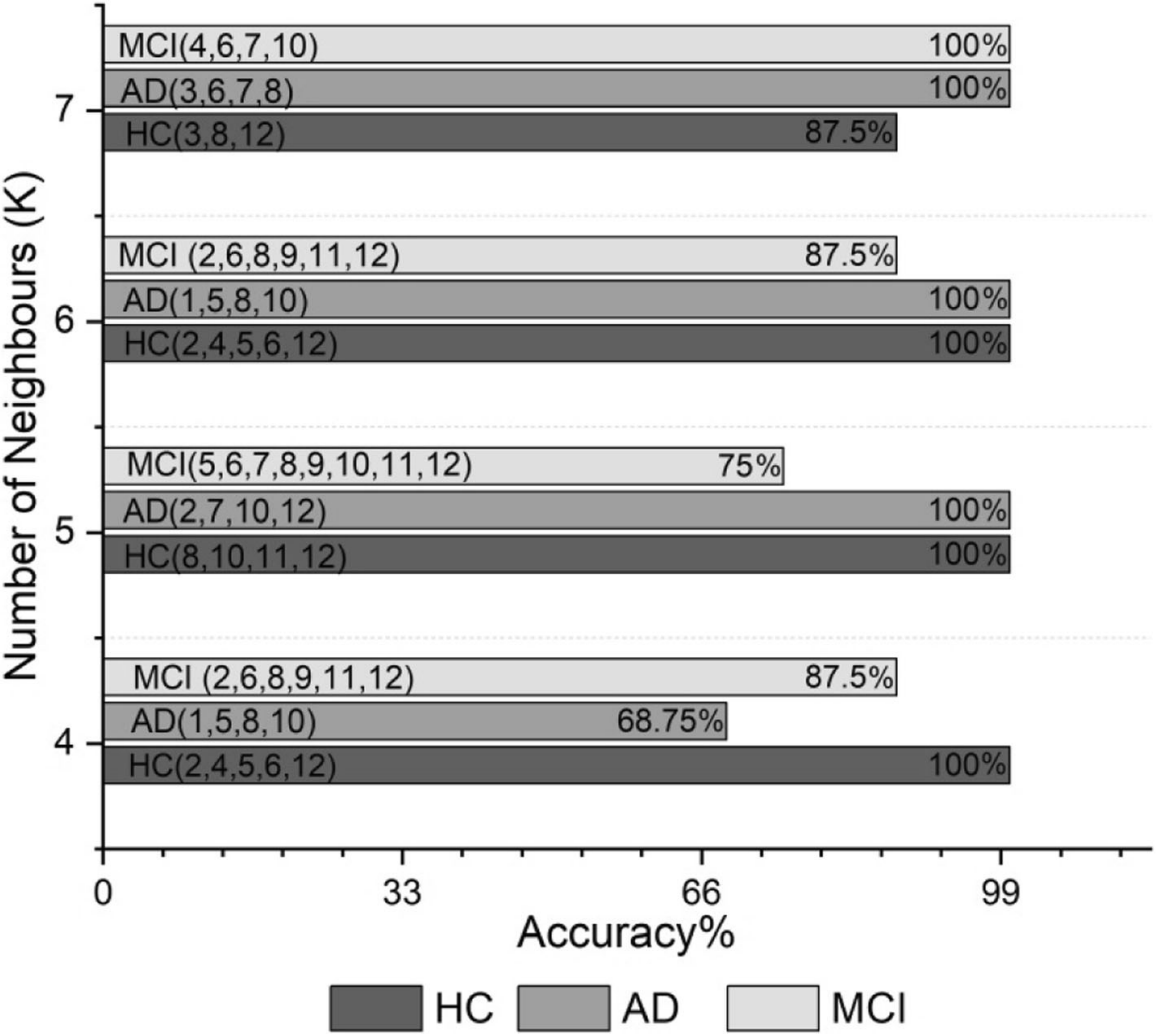
KNN accuracies for K = 4, 5, 6, 7

**Fig. 2:**
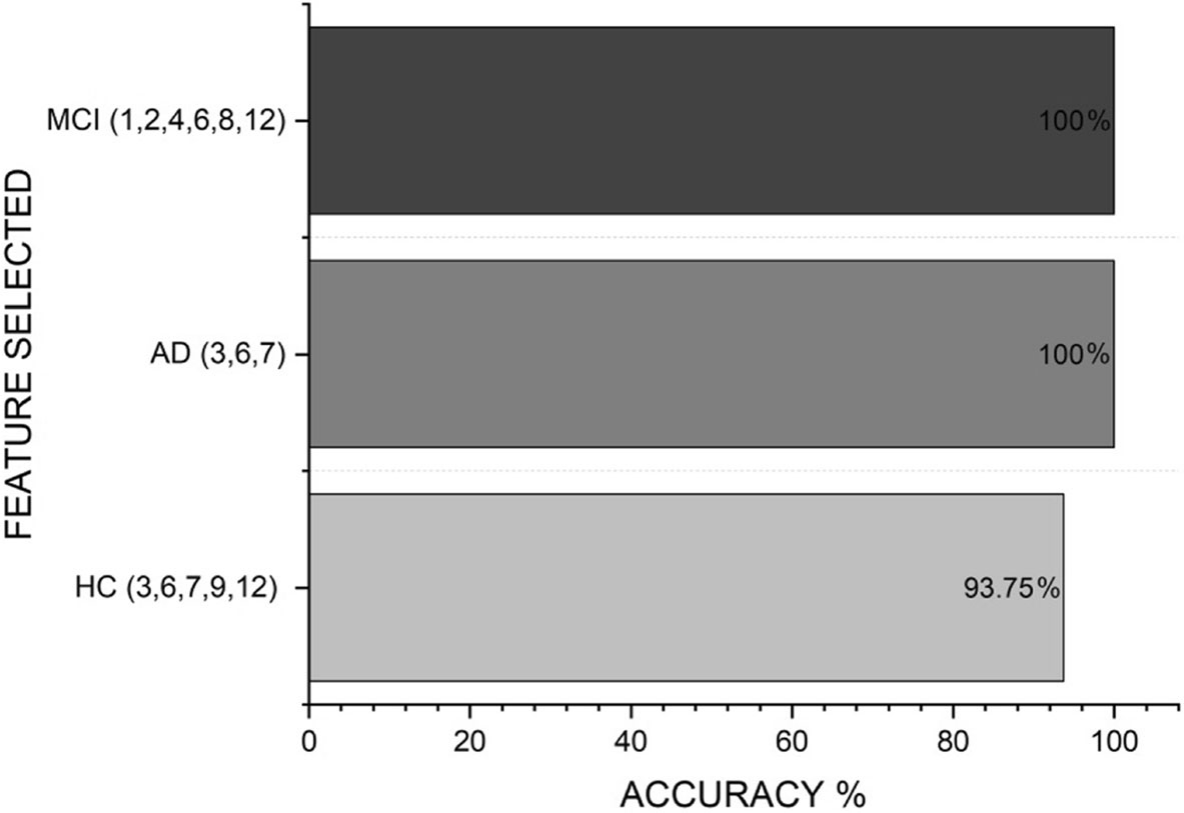
Accuracy of different classes using SVM polynomial order 3

**Fig. 3:**
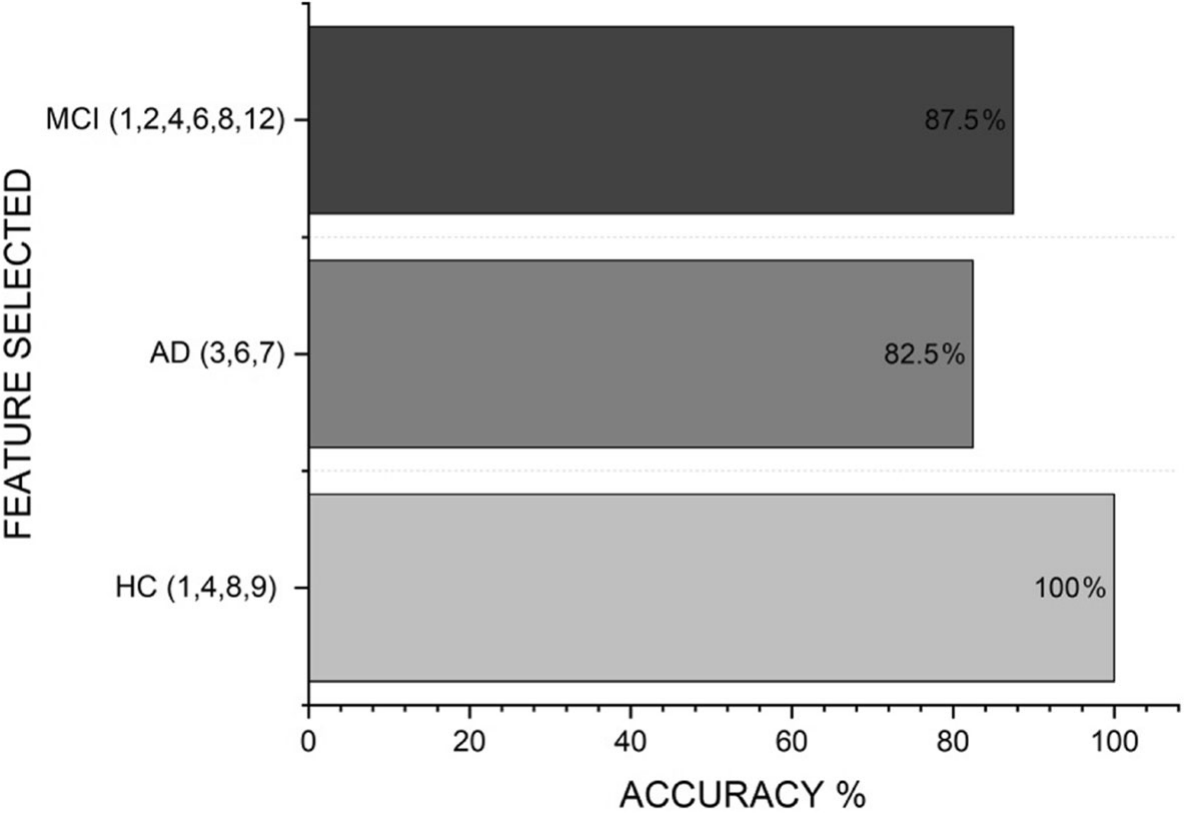
Accuracy of different classes using SVM polynomial order 4

The accuracy results achieved from the KNN and SVM classifiers are summarized in Table 3 and Figs. 1, 2 and 3.

**Table 3:** Accuracy of KNN and SVM classifiers

KNN		SVM	
Number of nearest neighbour	Accuracy	Polynomial order	Accuracy
4	85.42%	3	97.92%
5	91.67%		
6	95.83%	4	90%
7	95.33%

#### Inference

The study reveals that SVM has 97.92% accuracy with polynomial order 3. The selected features mean, contrast, kurtosis, and total brain area are the best selected features for recognizing normal cognition. Mean, entropy, contrast, homogeneity, kurtosis, and image symmetry are best used for identifying MCI. Energy, homogeneity, and skewness are optimal for identifying AD. KNN employs combinations of various features that are extracted from the image. With permutation for these features, 95.83% accuracy was obtained using KNN with K = 6 and K = 7. Thus, this study classified AD according to averaging of features selected from 10 slices rather than extracting all features extracted from 10 slices. Therefore, Gad et al. [100] proposed that these accuracies were the best compared to previous studies.

### Case study 2: SVM-based classification of neuroimages in AD: straight comparison of FDG-PET, rCBF-SPECT, and MRI data collected from the same individuals

#### Dataset

Ferreira et al. [20] analyzed 21 patients with mild AD and a group of 18 elderly HCs to present the investigation on the classification of AD for the multimodal technique that uses SVM in MRI and PET/SPECT images.

#### Methods

A whole-brain approach comprising a mask to eliminate voxels outside the brain has been employed. As a result, the feature vectors of 219727 voxels for all modalities have been obtained. Cerebellar normalization and global uptake normalization both have been utilized in this study [20, 103, 104].

SVM is applied [20, 26, 27, 105, 106] using the LIBSVM software [20, 107] (accessible in the PRoNTo toolbox) [20, 30] to differentiate HCs from patients with AD. The neuroimaging information was adjusted for the impact of age and education. Linear algebra operations including matrix transformation to eliminate confounding impacts from kernels using a residual matrix [20, 108, 109] was conducted, which showed that AD class were older and had fewer years of education than the control class. The dataset was partitioned into training and testing sets to estimate the generalization capability of the model. An estimate of the generalization error of the model can be made by repeatedly repartitioning the data. A leave-one-out cross-validation approach [20, 110, 111] has been used in which a single example (i.e., one patient with AD or HC) was left out of testing in each iteration.

#### Results

**Fig. 4:**
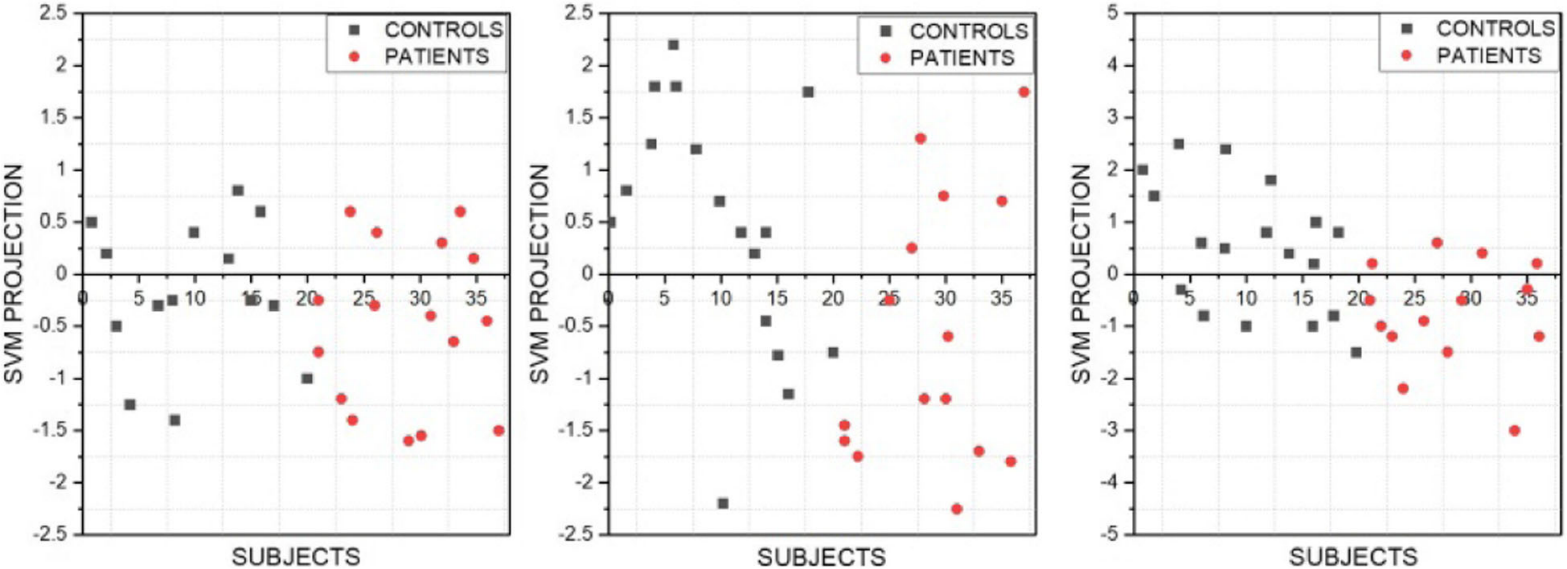
MRI PET and SPECT

Figure 4 illustrates the predictions for each single modality classification (MRI, FDG-PET, and rCBF-SPECT). Subjects classified as HCs are plotted below the horizontal line (i.e., with negative SVM projections), while subjects above the horizontal line presented positive SVM projections and therefore classified as having AD.

**Fig. 5:**
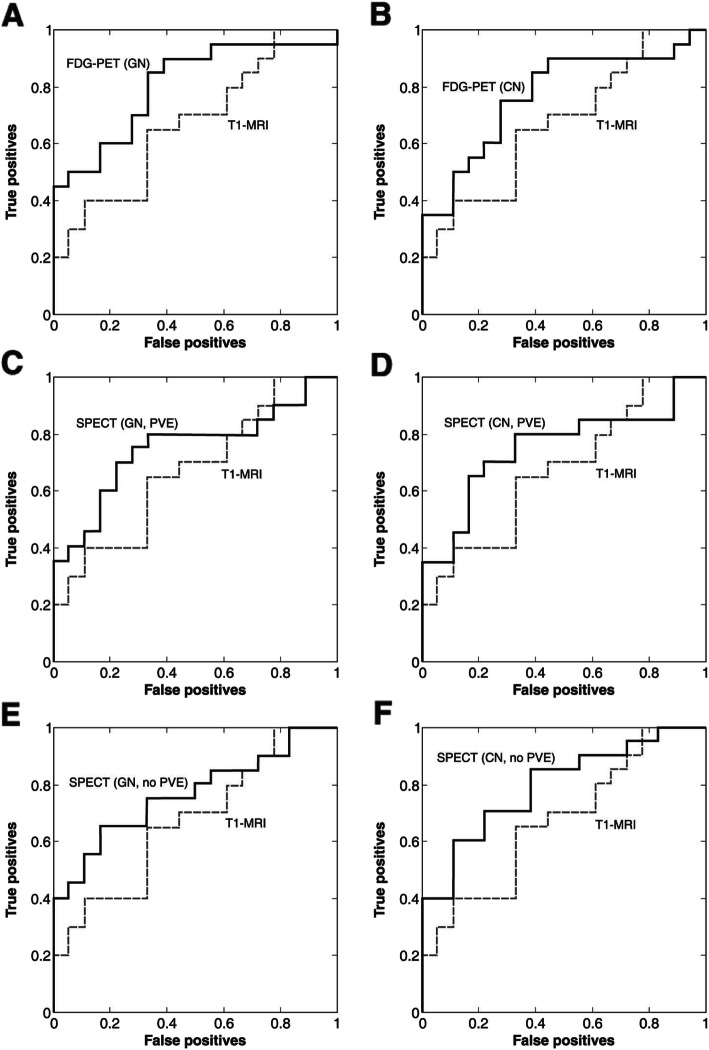
Receiver operating characteristic curves of classifications using a combination of imaging modalities

Figure 5 shows the receiver operating characteristic curves for each combination of modalities, and Table 4 shows the outcomes of investigations with a combination of modalities.

**Table 4:** Classification accuracy for individual and combined image modalities

	T-1 MRI	FDG-PET		rCBF-SPECT (GN)		rCBF-SPECT (CN)		T-1 MRI + FDG-PET		T-1 MRI + rCBF-SPECT (GN)		T-1 MRI + rCBF-SPECT (CN)	
		(GN)	(CN)	PVE	No PVE	PVE	No PVE	(GN)	(CN)	PVE	No PVE	PVE	No PVE
AUC	0.67	0.81	0.77	0.75	0.76	0.75	0.79	0.81	0.82	0.78	0.78	0.74	0.76
TP (%)	50.00	70.00	70.00	60.00	70.00	60.00	70.00	75.00	70.0	75.0	80.00	70.00	75.00
TN (%)	66.67	66.67	72.22	77.78	66.67	83.33	77.78	66.67	72.22	72.22	66.67	66.67	72.22
BA (%)	58.33	68.33	71.11	68.89	68.33	71.67	73.89	70.83	71.11	73.61	73.33	68.33	73.61
*p*-value*	0.1379	0.0242	0.0157	0.0210	0.0214	0.0071	0.0043	0.0198	0.0014	0.0008	0.0011	0.0253	0.0003

*BA* Balanced accuracy, *TN* True negative, *TP* True positive, *CN* Cerebellar normalization, *GN* Global normalization, *PVE* Partial volume effect correction

*Nonparametric statistical significance

The accuracy achieved using PET and SPECT data were the same. The PET accuracy rate was 68%–71% and AUC was 0.77–0.81, and the SPECT accuracy was 68%–74%, and AUC was 0.75–0.79. Moreover, both had better performance than analysis with T1-MRI data with an accuracy of 58% and AUC of 0.67. The inclusion of FDG-PET/rCBF-SPECT in MRI showed higher accuracy parameters (accuracy, 68%–74%; AUC, 0.74–0.82) than T1-MRI being used solely, but these were not better than the individual neurofunctional modalities.

#### Inference

Based on the observations, Ferreira et al. [20] proposed that FDG-PET and rCBF-SPECT produced better classification accuracies compared to MRI alone, and the addition of PET/SPECT to MRI provided higher accuracy.

## Challenges and future scope

Modern advancements and future scope of ML techniques offer encouraging applications in medical imaging. The use of imaging techniques, such as MRI, PET, and SPECT, has increased in the detection of inherent unfavorable effects of AD that initially might be clinically invisible because of the degree of patient’s cognitive impairment and confusion [112, 113]. Moreover, the challenge lies in finding the optimal method of integration of the imaging modality to generate the most effective diagnostic method for AD.

The application of SVM, KNN, and other ML algorithms demands a high level of technical expertise and clinical resources and knowledge. A huge acquisition of data-derived information is needed by a machine to function as an autonomous image interpreter [114], but several investigations have been conducted on only a small number of members, and it is therefore not reasonable to form specific conclusions on the diagnostic and prognostic significance of neuroimaging at the individual level.

In case of serious stages of AD, the treatment plan has to be decided promptly by a clinician. This aspect throws a great challenge to the application of SVM in neuroimaging as it can take several days to develop the reports because of some inherent SVM tasks, such as image preprocessing. Thus, it would be ineffective and potentially dangerous to patients to delay a clinical decision.

SVM would not be proper for those cases, which are recognized as having a gross neuroanatomical abnormality that is comorbid to their neurological disease [21]. Nevertheless, ML-based data processing methods could help shorten imaging time [115]. Further, such imaging methods could minimize unnecessary imaging, improve positioning, and assist in improvement of the characterization of the findings.

Another area in medical imaging where ML has an immediate impact is in the automated detection of findings. Moreover, CNN witnesses a great scope in the post-processing tasks of MRI, PET, and SPECT [116]. These tasks involve image registration, segmentation, and quantification. While SVM provides reliable classification of different classes, there are also probabilistic ML methods that provide an estimation of the probability that a provided data pertains to each category (e.g., 70% responder, 30% nonresponders) and hence propose to quantify the uncertainty in each prognostication. Gaussian processes [21, 117] and relevance vector machines [21, 118] are two such promising probabilistic classification methods that are currently used in applications in neuroimaging [21, 119, 120].

The application of regression methods to neuroimaging data has also shown a drastic development, which aimed to predict a continuous outcome (e.g., symptom severity) rather than a categorical class label. Certainly, pattern regression methods are presently being employed in neuroimaging data in health [121] and disease [122].

The essential benefit of such methods over traditional analytical procedures is that they enable conclusions to be made at the level of the individual and hence could be utilized in making treatment decisions in individual cases. Although there are remarkable theoretical and practical challenges in the application of ML approaches in neuroimaging, the results produced so far by various studies have proved to be encouraging toward the evolution of ML techniques for clinical detection, diagnosis, and prognosis.

## Conclusions

Various classification techniques for distinguishing patients with AD from HCs based on MRI findings have been evaluated in this study. According to the authors, the SVM classifier seems to manifest better accuracy rates when it comes to a clear margin of divisions among the classes compared to other classifiers. Moreover, it could provide reliable foresight about the test data due to the optimal boundary gap among the departing hyperplanes. SVM is more effective than other classifiers in the prediction of high-dimensional data due to its more limited parameter, making it operational and having high accuracy rates producing classifiers. Moreover, when it comes to dual problems, SVM manifest better results due to the utilization of kernel in solving the problem. As the risk of overfitting is less in SVM, it is more generalized. Lastly, SVM is also better as it can be employed for classification and regression problems.

To further increase the classification accuracy rates of the SVM classifier based on the single modality, that is, MRI, the addition of the PET/SPECT/CSF modality to MRI yielded better accuracy compared to MRI alone. Hence, according to the interpretation made from the study, the accuracy of the classifier is increased when a single modality is combined with other modalities. Multimodal approaches exhibit more relevant information, which is hidden when the single modality is examined, and they could display novel and different properties of the data by recognizing various correlations among the data.

## Data Availability

All relevant data and material are presented in the main paper.

## Abbreviations

AD: Alzheimer’s disease
MRI: Magnetic resonance imaging
MCI: Mild cognitive impairment
GPS: Global positioning system
ML: Machine learning
sMRI: Structural MRI
fMRI: Functional MRI
SPECT: Single-photon emission computed tomography
rCBF: Regional cerebral blood flow
PET: Positron emission tomography
SVM: Support vector machine
KNN: K-nearest neighbor
HCs: Healthy controls
SARs: Socially assistive robots
CSF: Cerebrospinal fluid
MMSE: Mini-mental state examination
CN: Cognitively normal
CAD: Computer-aided diagnosis
NCs: Normal controls
ADNI: Alzheimer’s disease neuroimaging initiative
MCIc: MCI convert
MCInc: MCI non convert
CNN: Convolutional neural network
DNN: Deep neural network
KSVMs: Kernel SVMs
OPLS: Orthogonal partial least squares to latent structures
NMF: Nonnegative matrix factorization
RS: Resting-state
AUC: Area under the curve
ROI: Region of interest
STAND: Structural abnormality index
